# Therapeutic Effect of Superficial Acupuncture in Treating Myofascial Pain of the Upper Trapezius Muscle: A Randomized Controlled Trial

**DOI:** 10.1155/2018/9125746

**Published:** 2018-12-02

**Authors:** Chao Ching Wang, Tse Hung Huang, Kuo Ching Chiou, Zi Yu Chang

**Affiliations:** ^1^Department of Traditional Chinese Medicine, Chang Gung Memorial Hospital, Keelung, Taiwan; ^2^School of Traditional Chinese Medicine, Chang Gung University, Taoyuan, Taiwan; ^3^School of Nursing, National Taipei University of Nursing and Health Sciences, Taipei, Taiwan; ^4^Graduate Institute of Health Industry Technology, Chang Gung University of Science and Technology, Taoyuan, Taiwan; ^5^Department of Finance, Chaoyang University of Technology, Taichung, Taiwan

## Abstract

The aim of this study was to compare the treatment efficacies of superficial acupuncture and traditional acupuncture on trigger points of the upper trapezius muscle. Forty people were recruited and randomly allocated to the traditional and superficial acupuncture groups. Each subject received two treatments per week in a four-week period. Outcomes were measured by visual analogue scale (VAS), the Northwick Park neck pain questionnaire scores (NPQ), and pressure pain threshold (PPT) assessments of trigger points. Data collected before the interventions were considered as baseline. Assessments were performed after the first treatment and at the end of the second and fourth weeks of treatment. Patients reported significant (p<0.05) and immediate improvements in VAS and PPT for both superficial acupuncture and traditional acupuncture after the first treatment and after two and four weeks. Significant improvements (p<0.05) in NPQ were attained after two weeks of treatments in both groups. Because superficial acupuncture is associated with less pain while producing immediate pain relief, we recommend it for treating myofascial pain syndrome in the upper trapezius muscle.

## 1. Introduction

Myofascial pain syndrome is a common source of musculoskeletal pain in primary care. About 30% of the patients who visit health care clinics because of pain meet the criteria for myofascial pain syndrome [1]. Commonly seen at muscles that are under prolonged eccentric loading [2], myofascial pain syndrome is characterized by trigger points, which are focal, discrete, and hyperirritable areas in taut bands of muscle fibers, are associated with typical pain referral patterns, and give rise to motor dysfunction and autonomic phenomena [3]. Because of its high prevalence and ease-of-access, the most commonly tested muscle is the upper trapezius muscle, which is implicated in neck and shoulder pain [4].

The formation of trigger points remains elusive. One hypothesis suggests that when excessive acetylcholine is released at the motor end plates, the calcium pump mechanism is disturbed, causing the sustained contraction of sarcomeres in the myofascial and local hypoxia due to blood vessel compression. This mechanism of action conforms to the energy crisis theory [5].

A variety of methods may be applied for treating myofascial pain. The noninvasive treatments include physical therapies, such as heat, massage, transcutaneous electrical nerve stimulations, stretching, and mud baths, or magnetic field application [6, 7]. The invasive methods include intramuscular electrical stimulation, injection therapy, and dry needling. Intramuscular electrical stimulation, or electroacupuncture, is usually performed with acupuncture needles as electrodes, the use of which provides more pain relief and improved functionality than traditional transcutaneous nerve stimulation [8]. Myofascial pain injections are performed with a variety of injectable drugs, such as procaine, lidocaine, isotonic saline solution, nonsteroidal anti-inflammatory drugs, corticosteroids, bee venom, botulinum toxin, or serotonin antagonists. However, Cummings and White concluded, “the nature of the injected substance makes no difference to the outcome, and wet needling is not therapeutically superior to dry needling.” [9, 10] Dry needling is an invasive technique in which needles are inserted into the skin and muscles, at the myofascial trigger points. The inserting and pistoning of the needles may elicit localized twitch responses, which may interrupt motor end-plate noise and reduce the pain [10]. The procedure of palpating the tender spots and inserting needles into them is similar to the traditional acupuncture technique of treating “Ah shi” points. The appearance of a localized twitch response is similar to the “de qi” sensation. Hong agreed that, with the way acupuncturists treat Ah shi points, they might well be treating myofascial trigger points [11]. Superficial acupuncture was first documented in “Huangdi Neijing” and was performed to treat nobles with good nutritional support who were characterized as having tender bodies by inserting small needles shallowly and leaving them inserted for a little time. Advocated by Baldry, superficial dry needling is a technique in which the therapist inserts an acupuncture needle into the tissues overlying each trigger point to a depth of 5-10 mm for 30 seconds. Practitioners claimed to have a successful practice, with minimal patient discomfort [12].

In this randomized controlled trial, we evaluated and compared the efficacies and adverse events of superficial acupuncture and traditional acupuncture for treating myofascial pain of upper trapezius muscle.

## 2. Materials and Methods

### 2.1. Design

This study is a parallel designed randomized controlled trial with the allocation ratio of 1:1, conducted at the Keelung branch of Chang Gung Memorial Hospital, Keelung City, Taiwan. It was approved by the Chang Gung Medical Foundation Institutional Review Board (IRB number 106-1472C1).

### 2.2. Subjects

Sample size calculation was based on a previous study testing the effect of dry needling on upper trapezius muscle [13]. Patients with neck and shoulder pain with VAS scores of two to seven, who were referred for evaluation and treatment from August 2016 to October 2017, were included. Physical examination revealed tender spots within palpable taut bands in the upper trapezius muscle, which showed a referred pain pattern when a pressure of 2.5 kgf/cm^2^ was applied to the taut bands [13].

Participants were excluded if they experienced any of the following criteria: (a) fibromyalgia, whiplash injury, cervical spine fracture, cervical radiculopathy, myeloid type cervical spondylopathy, and cervical spine surgery; (b) systematic disease such as rheumatoid arthritis, multiple sclerosis, or tuberculosis; (c) treatment or therapy for myofascial pain in the upper trapezius muscle within the past one month before the evaluation; (d) contraindications to acupuncture treatments, such as pregnancy, local infection, current anticoagulant therapy, and allergy to alcohol or stainless steel (Tables 1 and 2).

All participants were asked to stop any other treatment or therapy for myofascial pain syndrome while they were undergoing the acupuncture treatment and to report any adverse event they experienced. Randomization was performed by lots using opaque envelopes. Investigators ensured the envelopes were opaque when held to the light and opened after the participant's names were written on the envelopes.

### 2.3. Assessment and Follow-Up

**Figure 1:**
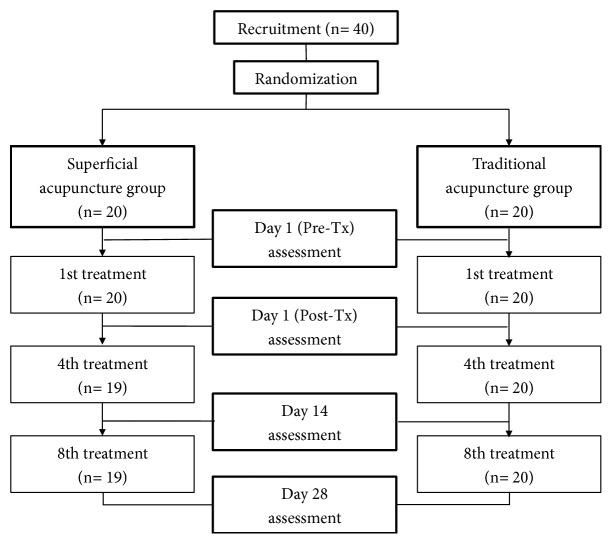
Flowchart of the study.

The VAS score, the Northwick Park neck pain questionnaire, and trigger point pressure pain threshold were assessed as outcome measures. The first assessment was performed before treatment at baseline. Assessments were then conducted immediately after the first treatment, at the end of the second and fourth weeks after treatment (Figure 1).

### 2.4. Measurement Instruments

The visual analogue scale is a psychometric response scale for subjective perception of current pain intensity, rated from 0 (no pain) to 10 (most severe pain ever experienced). The Northwick Park neck pain questionnaire has good short-term repeatability, high internal consistency, and sensitivity to change; it measures neck pain and consequent patient disabilities, providing an objective measure to evaluate the outcome and monitor symptoms in patients with acute or chronic pain over time [14, 15]. The participants had to fill in the questionnaire which was divided into nine five-part sections: (1) neck pain intensity, (2) neck pain and sleeping, (3) pins and needles or numbness in the arms at night, (4) duration of symptoms, (5) carrying, (6) reading and watching television, (7) working and/or housework, (8) social activities, and (9) driving. The assessments of pressure pain threshold were performed by the same licensed doctor using an algometer (model FDX 50 by Wangner Instruments, Greenwich, Connecticut, USA). Participants were asked to sit upright on a chair without armrests, with hip and knee joints flexed to 90°, with bilateral scapulae in a neutral position with both arms dropped naturally next to the trunk. The doctor explained the procedure to the participants at first and then marked the positions of trigger points in upper trapezius muscles. The algometer was perpendicularly applied to the skin surface of the trigger point, compressing the trigger point with a slow and steady force, which increased to approximately 1 kgf/cm^2^ per second. The participants were instructed to report “pain” once the participant felt any increase in pain intensity or discomfort. The doctor stopped applying force on the trigger point immediately and recorded the peak pressure showing on the algometer, expressed as kgf/cm^2^. Three repetitive measurements with an interval of 30 seconds were performed and averaged for pain threshold value [16].

### 2.5. Intervention

Participants were allocated randomly into superficial acupuncture or traditional acupuncture groups; they underwent treatment twice weekly for a 4-week period. During treatment, participants were asked to sit with both arms placed naturally alongside the body. A licensed doctor fully instructed the participants on the procedure and then gently wiped the trigger points on upper trapezius muscle with cotton swabs soaked with 75% alcohol.

#### 2.5.1. Superficial Acupuncture

Stainless steel ear acupuncture needles (0.2 mm diameter, 2.5 mm length. “YU KUANG” disposable ear acupuncture needles) were fully inserted perpendicularly into the skin over each trigger point with the depth of 2.5 mm. All needles were left in place for 20 minutes and then removed.

#### 2.5.2. Traditional Acupuncture

Stainless steel acupuncture needles (0.3 mm diameter, 40 mm length. “YU KUANG” disposable acupuncture needles) were inserted perpendicularly into the skin at each trigger point. The needles were inserted and withdrawn several times in each taut band using the pistoning acupuncture technique. One or more localized twitch responses were elicited. Before removal, the needles were inserted in the taut bands with an approximate depth of 1 cm for 20 minutes.

### 2.6. Statistical Analysis

Measurements of VAS, NPQ, and pressure pain threshold were calculated and presented as means ± standard deviations. The repeated measure ANOVA was used to assess the differences between the data before and after treatments in each group, and post hoc comparisons were performed using Fisher's least significant difference (LSD). Effect sizes were calculated and reported as eta square (*η*^2^). The statistical significance was established at p<0.05 (two-sided). SPSS version 22.0 for Windows was used for analyzing all data.

## 3. Results

The changes in parameters are listed in Table 3. No adverse event was reported by participants.

### 3.1. Reduced Pain Intensity

**Figure 2:**
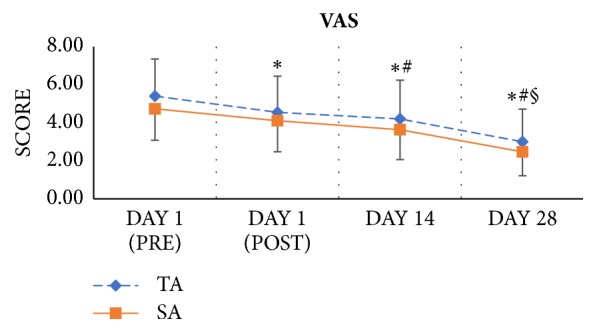
Serial changes in visual analogue score VAS. ^*∗*^*P*<0.05 versus DAY 1 (PRE); ^#^*P*<0.05 versus DAY 1 (POST); ^§^*P*<0.05 versus DAY 14. Error bars represent standard deviations.

According to the changes in VAS, all participants had reduced pain sensation in the upper trapezius muscle. Compared to the data collected at baseline, VAS scores after the first treatment and at the 2 and 4-week follow-up assessments showed significant improvements. A large main effect of time on pain intensity (p<0.001, *η*^2^ = 0.545) was observed. The results of both groups revealed that the pain intensity decreased as more treatments were applied (Figure 2). There was no significant time ^*∗*^ group interaction (p = 0.919, *η*^2^ = 0.003).

### 3.2. Increased Pressure Pain Threshold of Trigger Points

**Figure 3:**
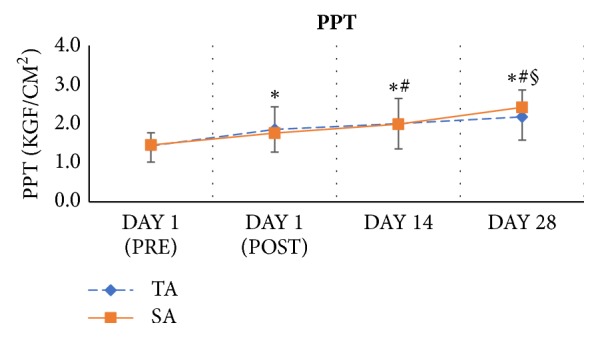
Serial changes in pressure pain threshold PPT, kgf/cm^2^. ^*∗*^*P*<0.05 versus DAY 1 (PRE); ^#^*P*<0.05 versus DAY 1 (POST); ^§^*P*<0.05 versus DAY 14. Error bars represent standard deviations.

The changes the in pressure pain threshold of trigger points revealed a significant objective relief of the myofascial pain syndrome after superficial acupuncture and traditional acupuncture treatments. A large main effect of time on pressure pain threshold of trigger points (p<0.001, *η*^2^ = 0.577) was observed. Pressure pain threshold increased as more treatments were applied to the participants in each group (Figure 3). No significant time ^*∗*^ group interaction (p = 0.136, *η*^2^ = 0.053) was shown.

### 3.3. Improvements in Neck Pain and Consequent Disability

The Northwick Park neck pain questionnaire is designed to show how patients' neck pain affects their ability to manage tasks in daily life. The NPQ score did not differ significantly before and after the first treatment in either group. However, at the 2- and 4-week follow-up assessments, significant improvements (p<0.01, *η*^2^ = 0.412) in NPQ scores occurred in both groups. The time ^*∗*^ group interaction was not significant (p = 0.852, *η*^2^ = 0.003).

**Figure 4:**
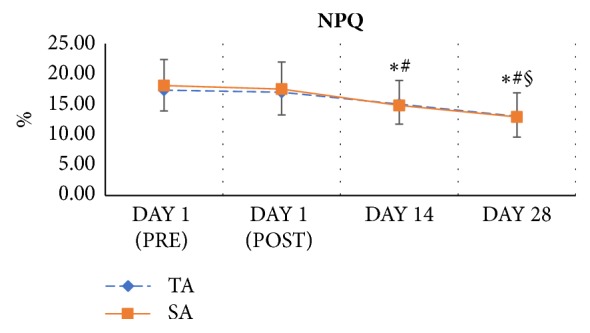
Serial changes of the Northwick Park neck pain questionnaire, NPQ. ^*∗*^*P*<0.05 versus DAY 1 (PRE); ^#^*P*<0.05 versus DAY 1 (POST); ^§^*P*<0.05 versus DAY 14. Error bars represent standard deviations.

The data indicate that the superficial and traditional acupuncture treatments improved the consequent disability due to neck pain after a series of treatments, but the treatments were not immediately effective (Figure 4).

## 4. Discussion

The aim of this randomized controlled trial was to evaluate the therapeutic effect of superficial acupuncture for treating myofascial pain syndrome in the upper trapezius muscle. Direct needling in the trigger point, as in Ah shi point acupuncture in Eastern medicine or dry needling in Western medicine, has been proven as an effective therapy for myofascial pain [9, 17–20]. In many cases, superficial acupuncture has been used as a sham control [21, 22]. However, many of the clinical trials could not differentiate the therapeutic effect of true acupuncture and superficial acupuncture. A systematic review of Moffet concluded that sham acupuncture could be as efficacious as true acupuncture [23], which implies a therapeutic effect for superficial acupuncture. In the early 1980s, Baldry suggested the technique “superficial dry needling” for treating myofascial pain syndrome by inserting the needle superficially into the skin and subcutaneous tissues overlying the trigger points [12, 24]. In contrast to deep dry needling, the advantages of superficial dry needling are relative painlessness, less risk of damage to nerves and blood vessels, minimal bleeding, and a low incidence of posttreatment soreness. Baldry advocated the use of superficial dry needling for treating primary nociceptive myofascial trigger point pain [25]. A review by Kalichman et al. suggested using the superficial needling technique over areas with potential risk of significant adverse events, such as lungs and large blood vessels [26]. Triggered by hyperglycemia, chronic inflammation, micro- and macrocirculatory dysfunction, hypoxia, autonomic and sensory neuropathy, and impaired neuropeptide signaling, diabetes can lead to a slowed healing response [27]. Acupuncture is contraindicated if the diabetes is unstable. With the advantages of less risk of damage to nerves and blood vessels, superficial acupuncture should be a feasible treatment option.

The exact mechanisms of action of trigger point dry needling remain elusive. Improving blood perfusion may ease the local hypoxia due to blood vessel compression, according to the energy crisis theory. Antidromic vasodilation is seen when unmyelinated C-fibers are stimulated. The neuropeptides released from peripheral terminals induce the neurogenic inflammation and increase the blood flow in skin and muscle [28, 29]. Sato et al. suggested the contribution of calcitonin gene-related peptide to antidromic vasodilation of skeletal muscle blood flow produced by acupuncture [30]. However, several reports have demonstrated the acupuncture-induced nitric oxide generation and increased local circulation [31–34]. Furthermore, Kimura et al. suggested that nitric oxide contributes to cutaneous vasodilation induced by acupuncture stimulation, whereas the antidromic vasodilation mediated by calcitonin gene-related peptide is less important [35]. A randomized, crossover experiment by Zhang et al. investigated the impact of acupuncture needle size and needling depth on microperfusion [36]. Superficial needling with thick needle (0.40X40mm, 2mm depth) induced the greatest blood perfusion changes compared with deep needling with thick needle (0.12X40mm, 1.5cm depth), and groups with piliform needle (0.12X40mm, 2mm or 1.5cm depth). While thick needle induced greater blood perfusion changes than piliform needle, there was no significant difference among needling depth. Goh et al. suggested that future acupuncture research should take into consideration the depth of insertion. In addition, bibliometric analysis should be used [37].

Mechanical disruption of the myofascial trigger point contraction knots may lead to changes in the end-plate cholinesterase and Ach receptors and contracture of cytoskeletal structures. When stimulated, A*δ* nerve fibers may activate enkephalinergic, serotonergic, and noradrenergic inhibitory systems. However, these mechanisms are induced only by deep needling to the trigger points but not by superficial needling [10]. Kawakita et al. proposed the polymodal receptors as possible candidates for acupuncture and moxibustion based on the facts that polymodal receptors are responsive to chemical, thermal, and mechanical stimulation, all of which induced the analgesic effect [38, 39].

There are several limitations to this study. First, the participants were not distributed evenly throughout the population. Women and young age predominated among participants, and these characteristics may be associated with the specific responses to acupuncture. Second, no placebo or control groups were included in this study design. The natural recovery from myofascial pain syndrome or the therapeutic effects of acupuncture could not be differentiated. Third, there was no follow-up after the 4-week treatment sessions. The long-term therapeutic effects could not be examined. Fourth, all treatments were performed by the author, bias in signaling to the patients could have occurred. Furthermore, well-designed and large-scale research studies are required to verify the results of this trial.

## 5. Conclusions

Both superficial acupuncture and traditional acupuncture showed immediate effects on reducing the sensation of pain and increasing the pressure pain threshold over the trigger points. The therapeutic effects increased as more treatments were applied to the trigger points during the 4-week session. Because superficial acupuncture is less painful and has minimal adverse effects, it is recommended for treating myofascial pain syndrome of the upper trapezius muscle.

## Figures and Tables

**Table 1:** Inclusion and exclusion criteria of the patients.

**Inclusion criteria**
(1) One or more tender spots within palpable taut bands over upper trapezius muscle
(2) Referred pain pattern is shown when a pressure of 2.5 kgf/cm^2^ was applied on the taut bands
(3) VAS of upper trapezius region within two to seven
**Exclusion criteria**
(1) Fibromyalgia, cervical radiculopathy, myeloid type cervical spondylopathy, and those who have had cervical spine operation
(2) A systematic disease such as rheumatoid arthritis, multiple sclerosis, or tuberculosis
(3) Therapy for myofascial pain in the upper trapezius muscle within the past one month before the evaluation
(4) Contraindication to undergoing acupuncture treatments, such as being pregnant, local infection, anticoagulant therapy, and allergy to alcohol or stainless steel

**Table 2:** Characteristics of study subjects.

Groups	Number	Sex M/F	Age mean ± SD
SA	19	5/14	33.37 ± 9.17
TA	20	9/11	35.35 ± 10.76
Total	39	14/25	

SA, superficial acupuncture; TA, traditional acupuncture.

**Table 3:** Baseline and follow-up characteristics by group.

Mean ±SD				
	Day 1	Day 1	Day 14	Day 28
	Pre-Tx	Post-Tx		

Superficial Acupuncture				

VAS	4.737 ± 1.661	4.105 ± 1.629	3.632 ± 1.571	2.474 ± 1.264
PPT	1.454 ± 0.438	1.759 ± 0.487	1.987 ± 0.634	2.419 ± 0.839
NPQ	23.17 ± 11.016	22.88 ± 11.544	15.56 ± 7.587	10.33 ± 7.831

Traditional Acupuncture				

VAS	5.40 ± 1.957	4.55 ± 1.905	4.20 ± 2.042	3.00 ± 1.717
PPT	1.433 ± 0.336	1.854 ± 0.579	2.000 ± 0.646	2.175 ± 0.685
NPQ	23.30 ± 14.020	22.22 ± 13.868	16.18 ± 10.166	11.34 ± 10.760

SD, standard deviation; Tx treatment; VAS, visual analogue scale 0-10; PPT, pressure pain threshold kgf/cm^2^; NPQ, the Northwick Park neck pain questionnaire, %.

## Data Availability

The data used to support the findings of this study are available from the corresponding author upon request.

## Supplementary Materials

Supplementary Materials CONSORT checklist of a randomized control trial.

## References

[1] Skootsky S. A., Jaeger B., Oye R. K. (1989). Prevalence of myofascial pain in general internal medicine practice. *Western Journal of Medicine*.

[2] Gerwin R. D., Dommerholt J., Shah J. P. (2004). An expansion of Simons' integrated hypothesis of trigger point formation. *Current Pain and Headache Reports*.

[3] Fernández-de-las-Peñas C., Alonso-Blanco C., Miangolarra J. C. (2007). Myofascial trigger points in subjects presenting with mechanical neck pain: a blinded, controlled study. *Manual Therapy*.

[4] Wallden M. (2014). The trapezius – clinical & conditioning controversies. *Journal of Bodywork and Movement Therapies*.

[5] Simons D. G., Travell J. G., Simons P. T. (1999). Travell & Simons' Myofascial Pain & Dysfunction. *The Trigger Point Manual.Volume 1. Upper Half of Body*.

[6] Pratsel H. G., Eigner U. M., Weinert D., Limbach B. (1992). The analgesic efficacy of sulfur mud baths in treating rheumatic diseases of the soft tissues. A study using the double-blind control method. *Vopr Kurortol Fizioter Lech Fiz Kult*.

[7] Vallbona C., Hazlewood C. F., Jurida G. (1997). Response of pain to static magnetic fields in postpolio patients: A double-blind pilot study. *Archives of Physical Medicine and Rehabilitation*.

[8] Ghoname E.-S. A., White P. F., Ahmed H. E., Hamza M. A., Craig W. F., Noe C. E. (1999). Percutaneous electrical nerve stimulation: an alternative to TENS in the management of sciatica. *PAIN*.

[9] Cummings T. M., White A. R. (2001). Needling therapies in the management of myofascial trigger point pain: a systematic review. *Archives of Physical Medicine and Rehabilitation*.

[10] Dommerholt J., del Moral O. M., Gröbli C. (2006). Trigger point dry needling. *Journal of Manual & Manipulative Therapy*.

[11] Hong C.-Z. (2000). Myofascial trigger points: pathophysiology and correlation with acupuncture points. *Acupuncture in Medicine*.

[12] Baldry P. (2002). Management of myofascial trigger point pain. *Acupuncture in Medicine*.

[13] Ziaeifar M., Arab A. M., Karimi N., Nourbakhsh M. R. (2014). The effect of dry needling on pain, pressure pain threshold and disability in patients with a myofascial trigger point in the upper trapezius muscle. *Journal of Bodywork and Movement Therapies*.

[14] Leak A. M., Cooper J., Dyer S., Williams K. A., Turner-stokes L., Frank A. O. (1994). The northwick park neck pain questionnaire, devised to measure neck pain and disability. *Rheumatology*.

[15] Yeung P. L. C., Chiu T. T. W., Leung A. S. L. (2004). Use of modified northwick park neck pain questionnaire in patients with postirradiation neck disability: Validation study. *Head & Neck*.

[16] Hsieh Y.-L., Kao M.-J., Kuan T.-S., Chen S.-M., Chen J.-T., Hong C.-Z. (2007). Dry needling to a key myofascial trigger point may reduce the irritability of satellite MTrPs. *American Journal of Physical Medicine & Rehabilitation*.

[17] Abbaszadeh-Amirdehi M., Ansari N. N., Naghdi S., Olyaei G., Nourbakhsh M. R. (2017). Therapeutic effects of dry needling in patients with upper trapezius myofascial trigger points. *Acupuncture in Medicine*.

[18] Gerber L. H., Shah J., Rosenberger W. (2015). Dry needling alters trigger points in the upper trapezius muscle and reduces pain in subjects with chronic myofascial pain. *PM&R: The Journal of Injury, Function, and Rehabilitation*.

[19] Kietrys D. M., Palombaro K. M., Azzaretto E. (2013). Effectiveness of dry needling for upper-quarter myofascial pain: a systematic review and meta-analysis. *Journal of Orthopaedic and Sports Physical Therapy*.

[20] Huang Y.-T., Lin S.-Y., Neoh C.-A., Wang K.-Y., Jean Y.-H., Shi H.-Y. (2011). Dry needling for myofascial pain: prognostic factors. *The Journal of Alternative and Complementary Medicine*.

[21] Wong E. L., Leung P., Zhang L. (2015). Placebo acupuncture in an acupuncture clinical trial. how good is the blinding effect?. *Journal of Acupuncture and Meridian Studies*.

[22] Näslund J., Näslund U., Odenbring S., Lundeberg T. (2002). Sensory stimulation (acupuncture) for the treatment of idiopathic anterior knee pain. *Journal of Rehabilitation Medicine*.

[23] Moffet H. H. (2009). Sham acupuncture may be as efficacious as true acupuncture: a systematic review of clinical trials. *The Journal of Alternative and Complementary Medicine*.

[24] Baldry P. (1995). Superficial dry needling at myofascial trigger point sites. *Journal of Musculoskeletal Pain*.

[25] Baldry P. (2002). Superficial versus deep dry needling. *Acupuncture in Medicine*.

[26] Kalichman L., Vulfsons S. (2010). Dry needling in the management of musculoskeletal pain. *Journal of the American Board of Family Medicine*.

[27] Baltzis D., Eleftheriadou I., Veves A. (2014). Pathogenesis and treatment of impaired wound healing in diabetes mellitus: new insights. *Advances in Therapy*.

[28] Uchida S., Hotta H. (2008). Acupuncture affects regional blood flow in various organs. *Evidence-Based Complementary and Alternative Medicine*.

[29] Gee M. D., Lynn B., Cotsell B. (1997). The relationship between cutaneous C fibre type and antidromic vasodilatation in the rabbit and the rat. *The Journal of Physiology*.

[30] Sato A., Sato Y., Shimura M., Uchida S. (2000). Calcitonin gene-related peptide produces skeletal muscle vasodilation following antidromic stimulation of unmyelinated afferents in the dorsal root in rats. *Neuroscience Letters*.

[31] Ma S.-X., Lee P. C., Anderson T. L., Li X.-Y., Jiang I. Z. (2017). Response of local nitric oxide release to manual acupuncture and electrical heat in humans: effects of reinforcement methods. *Evidence-Based Complementary and Alternative Medicine*.

[32] Shinbara H., Okubo M., Kimura K., Mizunuma K., Sumiya E. (2015). Contributions of nitric oxide and prostaglandins to the local increase in muscle blood flow following manual acupuncture in rats. *Acupuncture in Medicine*.

[33] Tsuchiya M., Sato E. F., Inoue M., Asada A. (2007). Acupuncture enhances generation of nitric oxide and increases local circulation. *Anesthesia & Analgesia*.

[34] Kimura K., Masuda K., Wakayama I. (2006). Changes in skin blood flow and skin sympathetic nerve activity in response to manual acupuncture stimulation in humans. *American Journal of Chinese Medicine*.

[35] Kimura K., Takeuchi H., Yuri K., Wakayama I. (2013). Effects of nitric oxide synthase inhibition on cutaneous vasodilation in response to acupuncture stimulation in humans. *Acupuncture in Medicine*.

[36] Zhang X., Park H.-J., Lee H. (2015). Do acupuncture needle size and needling depth matter? a laser doppler imaging study. *Integrative Medicine Research*.

[37] Goh Y. L., Ho C. E., Zhao B. (2014). Acupuncture and depth: future direction for acupuncture research. *Evidence-Based Complementary and Alternative Medicine*.

[38] Kawakita K., Shinbara H., Imai K., Fukuda F., Yano T., Kuriyama K. (2006). How do acupuncture and moxibustion act?—focusing on the progress in Japanese acupuncture research. *Journal of Pharmacological Sciences*.

[39] Kawakita K., Itoh K., Okada K. (2002). The polymodal receptor hypothesis of acupuncture and moxibustion, and its rational explanation of acupuncture points. *International Congress Series*.

